# Estimating the uncertainty of calculated out‐of‐field organ dose from a commercial treatment planning system

**DOI:** 10.1002/acm2.12367

**Published:** 2018-06-12

**Authors:** Lilie Wang, George X. Ding

**Affiliations:** ^1^ Department of Radiation Oncology Vanderbilt University Medical Center Nashville TN USA

**Keywords:** Eclipse treatment planning system, flattening‐filter free (FFF), Monte Carlo, out‐of‐field dose, TrueBeam

## Abstract

Therapeutic radiation to cancer patients is accompanied by unintended radiation to organs outside the treatment field. It is known that the model‐based dose algorithm has limitation in calculating the out‐of‐field doses. This study evaluated the out‐of‐field dose calculated by the Varian Eclipse treatment planning system (v.11 with AAA algorithm) in realistic treatment plans with the goal of estimating the uncertainties of calculated organ doses. Photon beam phase‐space files for TrueBeam linear accelerator were provided by Varian. These were used as incident sources in EGSnrc Monte Carlo simulations of radiation transport through the downstream jaws and MLC. Dynamic movements of the MLC leaves were fully modeled based on treatment plans using IMRT or VMAT techniques. The Monte Carlo calculated out‐of‐field doses were then compared with those calculated by Eclipse. The dose comparisons were performed for different beam energies and treatment sites, including head‐and‐neck, lung, and pelvis. For 6 MV (FF/FFF), 10 MV (FF/FFF), and 15 MV (FF) beams, Eclipse underestimated out‐of‐field local doses by 30%–50% compared with Monte Carlo calculations when the local dose was <1% of prescribed dose. The accuracy of out‐of‐field dose calculations using Eclipse is improved when collimator jaws were set at the smallest possible aperture for MLC openings. The Eclipse system consistently underestimates out‐of‐field dose by a factor of 2 for all beam energies studied at the local dose level of less than 1% of prescribed dose. These findings are useful in providing information on the uncertainties of out‐of‐field organ doses calculated by Eclipse treatment planning system.

## 1 INTRODUCTION

Therapeutic radiation to cancer patients is accompanied by unintended radiation to organs outside the treatment field. These out‐of‐field low doses that exist away from the primary radiation beams are from leakage and scatter. It is generally accepted that the low radiation dose level may contribute to secondary cancers in an individual's later life. The out‐of‐field dose during a radiation treatment course is one kind of this low‐level radiation and has been the topic of many investigations.^1^, ^2^, ^3^, ^4^, ^5^, ^6^, ^7^ Model‐based dose calculation algorithms are uncertain in predicting the out‐of‐field dose as they are not commissioned for that purpose.^8^ It is of great importance to know the out‐of‐field dose uncertainties calculated by using commercial treatment planning systems (TPS) as they are routinely used in patient radiotherapy treatment planning to evaluate doses to critical organs. Huang et al.^6^ performed an investigation to evaluate the accuracy of out‐of‐field dose calculations by the Pinnacle^3^ TPS for static IMRT treatment technique. Wang and Ding^7^ studied the accuracy of out‐of‐field dose calculations by anisotropic analytical algorithm (AAA) in the Eclipse TPS for the dynamic VMAT technique as well as the step‐and‐shoot IMRT technique for real clinical patient cases. Both investigations found that TPSs usually underestimate out‐of‐field doses. With the availability of flattening‐filter free (FFF) photon beams, studies have been carried out to find the impact on the out‐of‐field dose from the FFF beams.^2^, ^9^ It is found that the out‐of‐field dose is generally reduced for FFF beams and is dependent on patient parameters.^2^ These investigations on out‐of‐field dose from FFF beams are mostly based on phantom studies and were not focused on the evaluation of any TPS calculation accuracy for patient treatment planning. The objective of this study is to assess uncertainties of out‐of‐field doses calculated by a commercial treatment planning system in realistic patient treatment plans using photon beams of 6 MV, 6 MV‐FFF, 10 MV, 10 MV‐FFF, and 15 MV. Typical VMAT patient treatment plans for head‐and‐neck (H&N), lung, and prostate treatments are evaluated by using full Monte Carlo simulations in which the accelerator gantry rotation and MLC dynamic modulation are fully modeled for the incident beam.

## 2 MATERIALS AND METHODS

The commercial TPS evaluated in this study is Varian Eclipse Version 11.0 (Varian Medical Systems, Palo Alto, CA, USA) with AAA dose calculation algorithm. The out‐of‐field dose arises due to collimator scatter, patient scatter, and linac head leakage. A detailed Monte Carlo (MC) simulation of realistic modulated beams is an effective tool to study both treatment unit head leakage and phantom scatter accurately.^1^ The MC code used in this study is the EGSnrc^10^ (V4‐r2‐3‐2) code and its user codes BEAMnrc^11^ and DOSXYZnrc.^12^ The modulated realistic beams from the Varian TrueBeam linac with a Millennium 120 multileaf collimator (MLC) have been simulated by using BEAMnrc/DOSXYZnrc codes, and calculated dose distributions have been validated.^13^, ^14^ Varian TrueBeam phase‐space files^15^ (version 2.0) are used as the radiation sources at the plane before entering the JAWS and MLC. All five photon energies, both flattened and FFF beams (6X, 6FFF, 10X, 10FFF, and 15X), of a TrueBeam linac are investigated in this study. The JAWS openings and MLC modulations are modeled in detail in the simulations as described in the study by Lobo and Popescu.^16^ The typical source phase‐space file for each beam energy used for simulation is about 40 GB in size, containing about 1.8 billion particles. The out‐of‐field dose is defined in this study as the dose in areas where the dose level is less than 5% of the prescribed target dose. Unless otherwise specified, the calculation grid size for AAA algorithm in Eclipse is 5 mm and the voxel size for MC calculation is also 5 mm. The 5‐mm voxel size is justified because the out‐of‐field dose is usually in areas far from the regions where high‐dose gradients are present. More importantly, a larger voxel size gives better statistics for MC calculations. For the comparison of the out‐of‐field dose distributions for the two calculation algorithms, both water phantom and CT‐image‐based calculations were performed.

### 2.A Phantom study

In a previous study,^7^ we reported the out‐of‐field dose from a standard 6‐MV beam from a Varian Clinac accelerator; we extend this study to include FFF beams and higher energy photon beam from a TrueBeam linac. The out‐of‐field dose distributions calculated by Eclipse are evaluated in a water phantom (40 × 40 × 40 cm^3^) by comparing results between Eclipse and MC calculations for a static field of 4 × 4 cm^2^ for 6 MV FFF, 10 MV FFF, and 15 MV photon beams. Three scenarios are considered: (a) field shaped by MLC with jaw openings at 20 × 20 cm^2^; (b) field shaped by jaws with MLC parked; (c) field shaped by MLC and jaws both setting at 4 × 4 cm^2^. The gantry and the collimator angles were set to 0 with source‐to‐surface distance (SSD) = 100 cm.

### 2.B CT‐based patient dose calculations

For CT image‐based patient dose calculations, the same patient CT images and the beam parameters including jaw settings and MLC modulation sequences were used in MC simulations. Four materials (air, lung, tissue, bone) were employed in converting CT number to material and density. The statistical uncertainties of the MC calculations are less than 1% for calculated voxels inside the treatment field. Three realistic cancer patient treatment plans were investigated which included different photon beam energies from the TrueBeam linac and involved different treatment sites: H&N, lung, and prostate. All three treatment plan cases used the VMAT technique with fixed jaw settings, that is, jaw tracking feature for TrueBeam in Eclipse was turned off in the VMAT optimizations as we have not yet implemented jaw tracking capability for MC calculations. For H&N treatment plans, the comparisons were performed between MC and Eclipse for both 6 MV and 6 MV‐FFF beams with the same MU settings, respectively. Similarly, for the lung case, both 10 MV beam and 10 MV‐FFF beam are investigated. For prostate treatment plans, we studied out‐of‐field doses for 15 MV beams in addition to 10 MV and 10 MV‐FFF beams. Note that the plans used for treating patients were produced with specific photon energy, following the institution's clinical procedures; however, for the purpose of this investigation, we studied the effects of using different photon energies. For example, the lung patients are normally treated with 6 MV photon beam; however, 6 MV‐FFF, 10 MV and 10 MV‐FFF beams have also been used especially for large patients.

## 3 RESULTS

### 3.A Out‐of‐field dose in water phantom

**Figure 1:**
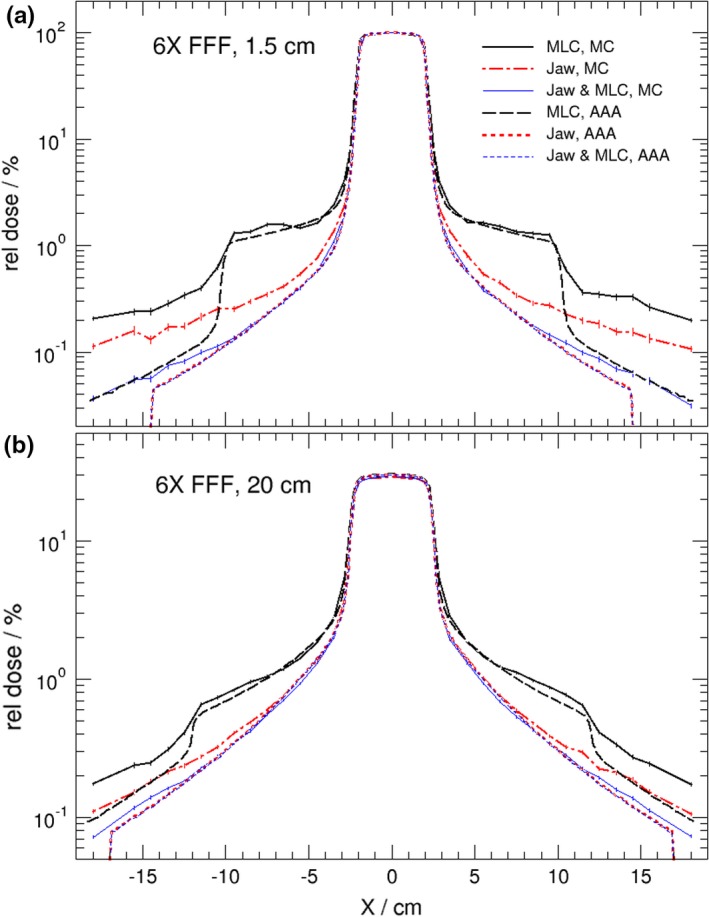
Lateral dose profiles comparison of Eclipse (AAA) and MC at two different depths (1.5 cm, 20 cm) in water phantom for static 4 × 4 cm^2^ 6 MV FFF beams from a TrueBeam linac. Field size of 4 × 4 cm^2^ is shaped by (a) MLC with jaws opening at 20 × 20 cm^2^; (b) jaws only with MLC retracted, and (c) both MLC and jaws at 4 × 4 cm^2^. Doses are normalized on the central axis at 1.5 cm depth.

**Figure 2:**
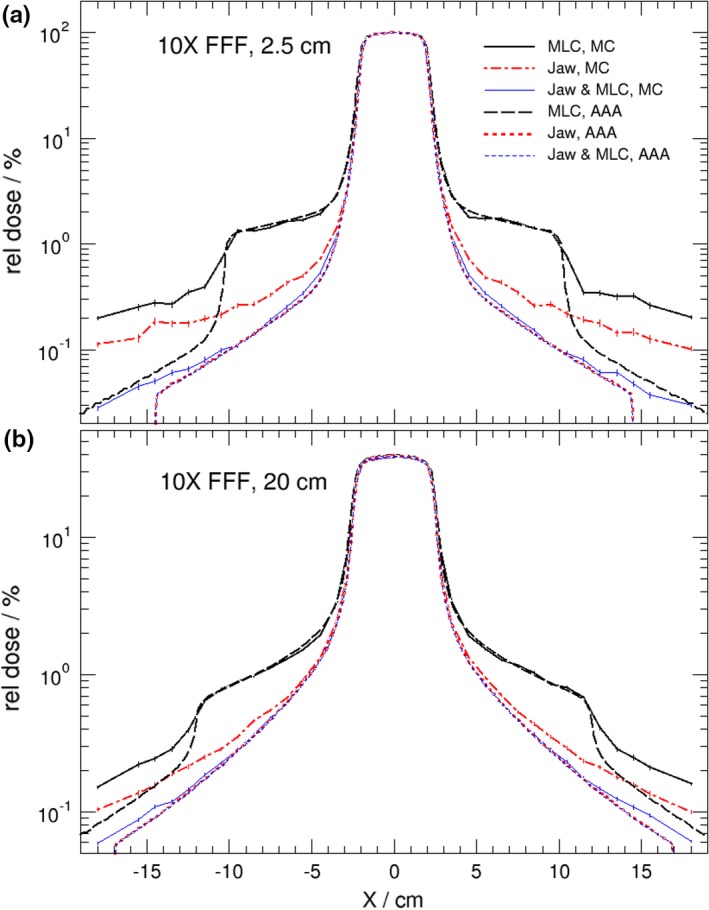
Lateral dose profiles comparison of Eclipse (AAA) and MC at two different depths (2.5 cm, 20 cm) in water phantom for static 4 × 4 cm^2^ 10 MV FFF beams from a TrueBeam linac. Field size of 4 × 4 cm^2^ is shaped by (a) MLC with jaws opening at 20 × 20 cm^2^; (b) jaws only with MLC retracted, and (c) both MLC and jaws at 4 × 4 cm^2^. Doses are normalized on the central axis at 2.5 cm depth.

**Figure 3:**
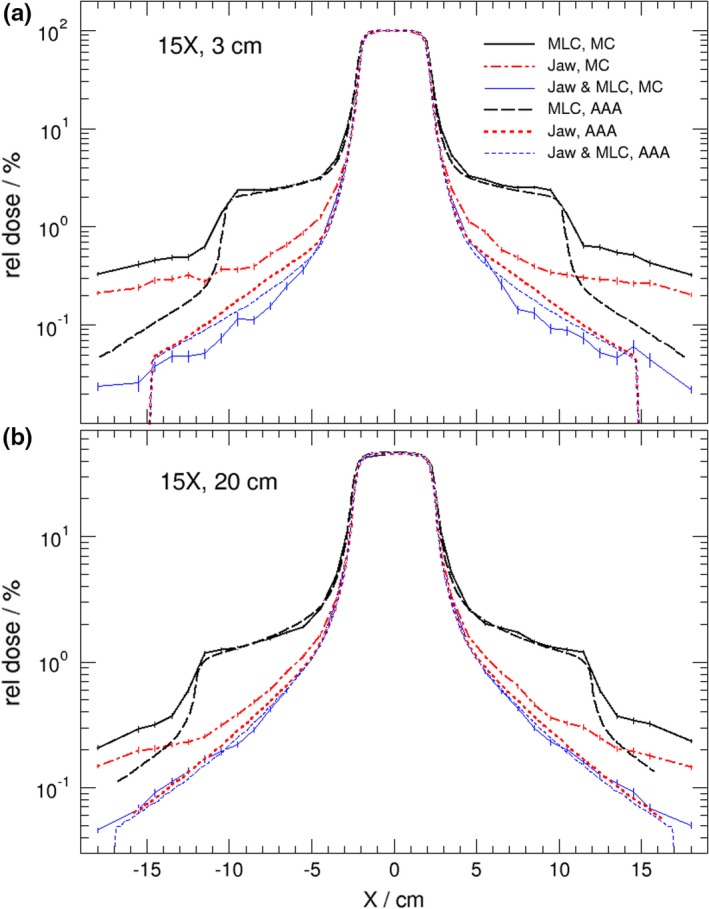
Lateral dose profiles comparison of Eclipse (AAA) and MC at two different depths (3 cm, 20 cm) in water phantom for static 4 × 4 cm^2^ 15 MV beams from a TrueBeam linac. Field size of 4 × 4 cm^2^ is shaped by (a) MLC with jaws opening at 20 × 20 cm^2^; (b) jaws only with MLC retracted, and (c) both MLC and jaws at 4 × 4 cm^2^. Doses are normalized on the central axis at 3 cm depth.

Figure 1 shows the calculated dose profile comparisons between AAA and MC for the 4 × 4 cm^2^ 6 MV FFF beam irradiations at (a) a depth of maximum dose, and (b) a 20 cm depth in the water phantom. The results presented in Fig. 1 are very similar to those of the previous study^7^ for a Varian Clinac 6 MV flattened beam in that the AAA underestimates out‐of‐field dose by a fraction of a percent compared with MC calculations and that the difference between AAA and MC becomes smaller at deeper depths. As before, the MC calculated out‐of‐field dose level is independent of depth, but the AAA gives a higher dose level at deeper depths which makes the discrepancy smaller. Very similar results are observed for 10 MV‐FFF and 15 MV photon beams from the Varian TrueBeam, respectively, as shown in Figs. 2 and 3. A noticeable result from Figs. 1–3 is that the differences in calculated out‐of‐filed doses between Eclipse and MC were reduced when the JAWS were set to 4 × 4 cm^2^ as well. This is also true for standard 6 MV photon beam (not shown). This result indicates that accuracy of Eclipse predicted out‐of‐field dose improves when fields are shaped by both jaws and MLC.

### 3.B CT‐based patient dose calculations

**Figure 4:**
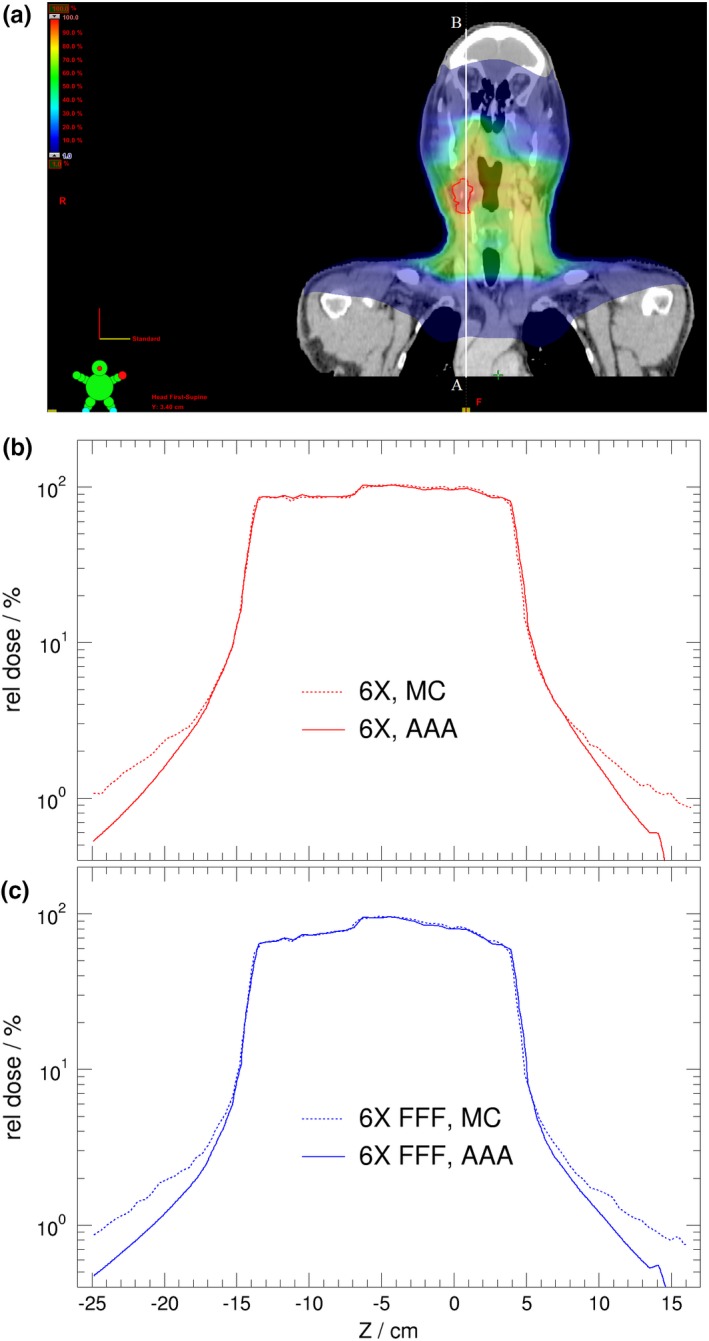
The dose distribution for H&N case treated with VMAT technique (a). Lateral dose profile calculated by AAA and MC, using photon beam energy of either 6 MV (b) or 6 MV FFF (c). The dose profiles are indicated by the white line from A to B in (a). The ordinate is shown in logarithm scale.

**Figure 5:**
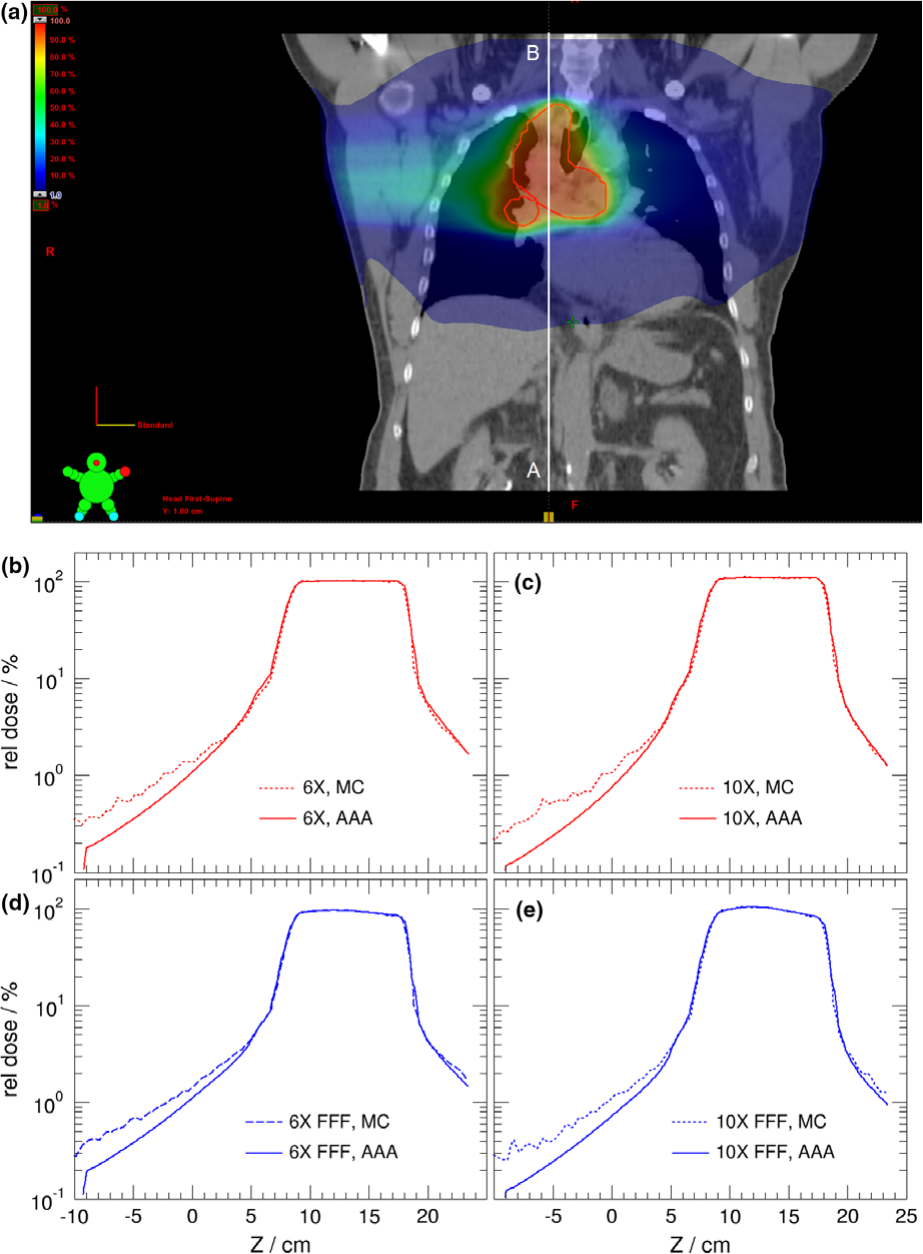
The dose distributions for lung treatment plan treated with VMAT (a). Dose profiles are calculated by Eclipse (AAA) and MC using photon beam energies of 6 MV (b), 10 MV (c), 6 MV FFF (d), and 10 MV FFF (e). The dose profiles are indicated by the white line from A to B in (a). The ordinate is shown in logarithm scale. MC dose is normalized such that MC and AAA for the corresponding beam have the same mean target dose.

**Figure 6:**
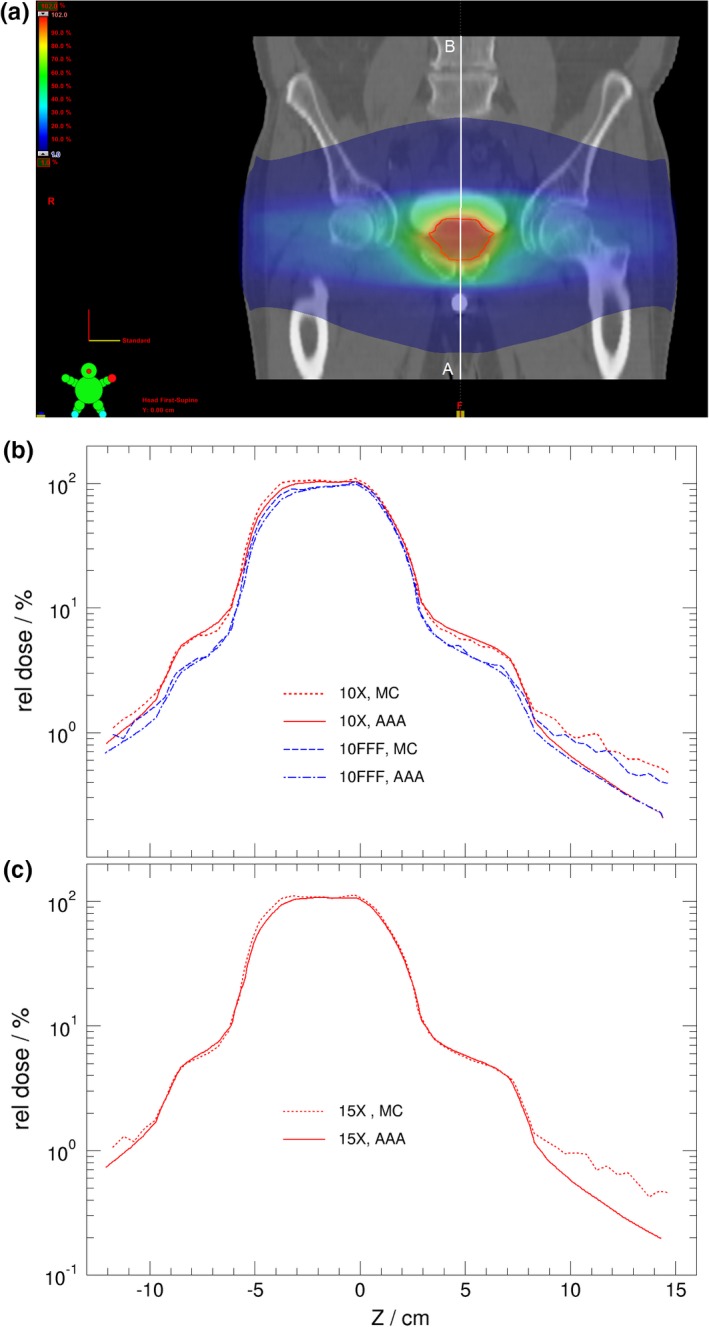
The dose distribution for prostate plan treated with VMAT (a). Dose profiles are calculated by Eclipse (AAA) and MC using photon beam energies of 10 MV and 10 MV‐FFF (b), and 15 MV (c). The dose profiles are indicated by the white line from A to B in (a). The ordinate is shown in logarithm scale.

Figures 4, 5, 6 present dose comparisons between Eclipse and MC for H&N, lung, and prostate patient treatment plans, respectively. The dose shown in color‐wash starts at 1% dose level. This dose level corresponds to typical leakage radiation from linac head. The dose profiles are shown in superior–inferior direction. It is seen in Fig. 4, the AAA underestimates out‐of‐field dose at dose levels of 1% of the prescription dose by about 30~50% of local dose compared with that of Monte Carlo calculations which correspond to 0.4~0.5% of the prescription dose in the superior region (brain) and about 0.7~0.8% of the prescription dose in the inferior region (lung), for both standard 6 MV and 6 MV‐FFF beams. There is a small improvement for calculated out‐of‐field dose estimation for the 6 MV‐FFF beam compared with that of standard 6 MV beam at the same coordinate Z position. This observation is consistent with the reported result that out‐of‐field dose is generally reduced for flattening‐filter free beams.^2^, ^9^

For the lung treatment plan shown in Fig. 5, the comparison of dose profiles are done for both standard (6 MV, 10 MV) beams and FFF (6 MV‐FFF, 10 MV‐FFF) beams between Eclipse and MC. It is seen that Eclipse underestimates dose by 40%–50% of local dose compared with that of Monte Carlo calculations at dose level of 1% of the prescription dose independent of beam energies for both standard and FFF beams. As before the Eclipse calculations underestimated out‐of‐field dose by a factor of 2 in real patient treatment planning.

Similar results are seen in Fig. 6 for the prostate plan where Eclipse underestimates dose by about 30%–50% of local dose compared with that of Monte Carlo calculations at dose level of 1% for both standard 10 MV and 10 MV‐FFF as well as 15‐MV photon beams. The out‐of‐field dose for FFF beam is reduced insignificantly compared with that for the beam with flattening filters. There is little difference observed between using photon energies 10 MV and 15 MV. It is worth noting that the VMAT optimizations in Eclipse were done with JAW tracking option turned off in all patient treatment plans studied here. The discrepancy between Eclipse and MC may be less with JAW tracking option turned on as indicated by results shown in previous Section III.A.

Table 1 presents examples of the comparison of mean doses, between AAA and MC calculations, for some typical organs outside of the treatment field for the three clinical cases studied. Dose levels at these organs are all below 2% of prescription doses. As expected, the Eclipse calculated out‐of‐field organ doses are consistently underestimated by 0.2~0.4% of the prescription dose, independent of treatment sites and photon beam energies.

**Table 1:** Calculated mean doses as percentage of the prescribed dose in organs outside of the treatment field for the head‐and‐neck (H&N), the lung and the prostate cancer patients. Dose comparison is made between AAA and MC calculation methods and there is the same mean PTV in both MC and AAA calculations

Organs	AAA (% of prescribed dose)	MC (% of prescribed dose)
H&N 6 MV		
Eye	1.8%	2.0%
Heart	0.6%	1.1%
Lung 10 MV		
Kidney	0.1%	0.3%
Liver	0.5%	0.9%
Prostate 15 MV		
Kidney	0.4%	0.7%

## 4 CONCLUSIONS

Using Monte Carlo simulations, we evaluated the accuracy of the out‐of‐field dose predicted by Eclipse system (V.11) with AAA algorithm for a Varian TrueBeam linac for photon beam energies ranging from 6 to 15 MV beams including flattening‐filter free beams as well. The Eclipse calculated out‐of‐field dose distributions were compared with those of Monte Carlo calculated in both phantoms and in realistic patient CT‐based treatment plans. For all beam energies studied, the Eclipse system consistently underestimates out‐of‐field dose by 30~50% of local dose (with jaw tracking turned off) compared with that of Monte Carlo calculations. These underestimations occur at locations where local doses are less than 1% of the prescription dose. These findings are useful in providing information on the uncertainties of out‐of‐field organ doses calculated by Eclipse treatment planning system. The more accurate out‐of‐field doses can be estimated by multiplying Eclipse calculated doses by a factor of 2 at the local dose level of 1% or less of prescribed dose.

## CONFLICT OF INTEREST

The authors have no conflict of interest to disclose.
